# Proceedings of the Central New York Homœopathic Medical Society

**Published:** 1888-03

**Authors:** Julius G. Schmitt

**Affiliations:** Secretary; Rochester, N. Y.


### PROCEEDINGS OF THE CENTRAL NEW YORK HOMŒOPATHIC MEDICAL SOCIETY.

ROCHESTER, N. Y., December 15th, 1887.

Dr. T. D. Stow, the President, called the meeting to order at eleven A. M. The following members answered to the roll-call: Drs. T. D. Stow, J. A. Biegler, C. W. Baker, J. B. Voak, A. J. Brewster, W. F. Clapp, W. A. Hawley, R. A. Adams, Allen B. Carr, J. G. Schmitt. Drs. A. V. Hoard and R. C. Grant, of Rochester, were present as visitors and invited to participate in the discussion.

The proceedings of the previous meeting were read by the Secretary and approved.

Dr. T. D. Stow was gratified to see so many members present, and thanked them for the honor conferred upon him by his election as President for the coming year.

Dr. Hawley, as Chairman of the Board of Censors, reported that there were no applications for membership, as the application of Dr. True had not been made in proper form, according to the new Constitution, but would report on him at the next meeting. In the meantime, application of Dr. R. C. Grant had been handed in, which was reported to the Society.

Dr. Stow read section 73 from the *Organon*, treating the subject under discussion.

Dr. Hawley—One question is suggested by this article, namely: Is it to be supposed that when an epidemic has assumed the same form, we have to treat all patients attacked by it the same way as the old school does?

Dr. Stow—Hahnemann's foot-note to section 73 covers this ground.

Dr. Brewster—There are complications in epidemics which are not essential to the disease. These are mostly caused by the latent psora, now aroused into action, and require specific treatment. Epidemic remedies may cover ordinary cases, but will not correspond with the complications. I do not believe in "sheet-anchors," as I once heard a physician express himself, when speaking of biniodide of Mercury as the "sheet-anchor" in diphtheria.

Dr. Schmitt—I remember a case of measles, at a time when Belladonna cured most of the cases, where the eruption was only partially developed, and Calcarea carb., indicated by the constitutional symptoms of the child, brought it out fully and cured it.

Dr. Brewster—A case of measles complicated with convulsions, which came on every six hours regularly, Ignatia cured. Indications: First, *regularity* in the appearance of the convulsions; second, *dazed, paralyzed* condition of the patient after the convulsions; and, third, *sudden changes of temper*, from great pleasantness to real ugliness.

Dr. Clapp—What was the cause of the convulsions? The only case I remember ever having lost of measles was the following: A woman, twenty-seven years old, was taken with the measles. Bryonia given according to indications brought out the eruption, and she was doing so well the next day that I stopped attendance. Twelve hours later I was called in great haste, messenger reporting her dying. When reaching the house, which was an hour's ride distant from my office, I found her breathing her last. Shortly before death, hemorrhage from all the apertures of the body, eruption having turned blue two hours before. There must have been something more than the measles in this case.

Dr. Brewster—In answer to Dr. Clapp, I think that the poison of the measles irritated the nerve centres of the child, and thus caused convulsions.

Dr. Hawley—We must expect variations in all these diseases, resulting from the difference in the individualities. Further we cannot go. There seem to be, oftentimes, idiosyncrasies which modify such diseases. Ordinary measles will pass through safely without any medicine. I never lost a case of measles as long as I can remember. If Dr. Clapp had come an hour sooner, Crotalus, Lachesis, or some such remedy might have done something.

Dr. Voak—In complications of epidemic diseases I am inclined to throw in a dose of Sulphur $^{2c}$ or $^{55m}$. I have no remedy for measles, but treat the patient.

Dr. Clapp—Ordinary cases of epidemics will get along without medication, and I give to my families instructions in reference to simple measles, as to regimen and so on, so that they need not call on me.

Dr. Schmitt—There seems to be a tendency with physicians, that the older they grow the less medicine they give. I have come to the point that I prescribe Saccharum lactis rather than medicine for a simple, uncomplicated case of any disease.

Dr. C. W. Baker—When graduating in Chicago, I thought I was a homœopath, until I found out I had learned very little of it there. That time I gave the third decimal trituration because I imagined that by going higher I was not giving any medicine at all. Now I give very little medicine and I am well satisfied with the result following this small dosage. I was called to treat a cough, which was so incessant that the patient could hardly utter a word between the paroxysms. This had lasted over six hours. One dose of Phosphorus $^{cm}$ put her into a sound sleep within half an hour.

Dr. Grant—It has been a serious question with me how much we really do with medicine. I am getting afraid of our remedies, as they invariably will aggravate the case if not properly chosen. I lost one case of measles last winter where there was a complication with membranous croup.

Dr. Hawley—I should shake in my boots for fear of having my record in measles spoiled if I should find such a complication. I have made a rule to keep my patients, whether they are suffering from measles or scarlet fever, in the bed until the time of desquamation is over, and do refuse to treat cases where my orders are not obeyed, and I think that the enforcement of this rule is the keynote to my success.

Dr. Grant—I treated a case of diphtheria last summer in a child seven years old; there was a thick yellow coating on both tonsils .and in the pharynx up to the posterior nares. The tongue was coated with a heavy yellow fur; stringy discharge from both nostrils. One dose of Kali bichrom.$^{2c}$ was given. Now, Guernsey says that a case treated with the single dose of the right remedy will never have sequelæ. This case progressed favorably and was discharged after three days. After the lapse of three days more, I was sent for in a hurry. I found the patient white, pale, very restless, thirsty, suppression of urine for twenty-four hours; change came at midnight; heart in a fluttering condition. Arsenic $^{2c}$, one dose, was given and instruction left not to raise the patient from her recumbent position. She died a few hours later, while she was raised very carefully to urinate.

Dr. Hawley—I have had such experiences. I think the remedies had been selected correctly, but the complete loss of vitality caused the formation of heart-clot.

A recess was taken until half-past two P. M. After the recess Dr. Stow read a paper on "Contagious Diseases, Caused by Acute Miasmata, According to Section 73 of the *Organon*."

(Dr. Stow's paper was given in our February issue.)

Dr. Hawley—This paper contains some very interesting points. The ordinary notions of propagation of contagious diseases are untenable. Dr. Stow has come nearer the cause than anything I have heard before. Whatever causes epidemic cannot be measured nor seen by the microscope. While I was a student in Albany, a fellow-student and myself dissected a body under demonstrator Dr. Swinburne late in the fall. In November I came down with varioloid and my fellow-student with confluent small-pox. There were no other cases of small-pox in Albany. My fellow-student got well, went then to Stephentown, and after being there three weeks, a pustule which had formed under his nail he opened by drilling through the nail. He took all proper precaution to clean the instrument and his hands, then he rode eight miles in the country and stopped at a house to deliver a verbal message, without touching anybody, not even leaving his horse, and just twelve days later the lady to whom he had delivered the message came down with variola, and she was the only one that had it. Now, I tried to find out how I got the varioloid and my friend the small-pox, and it turned out, that while we came in contact with Dr. Swinburne, he treated a child having the small-pox. This occurrence impressed upon me the thought that contagion is not caused by a germ, but by a dynamic force, and that the subject upon which this force acts, must have a peculiar tendency to succumb to its influence and this only corroborates our theory of the dynamization of our remedies. Take an empty phial which has contained once medicated globules and fill it with unmedicated globules and they will become medicated.

Dr. Voak—I have always maintained that contagious diseases can only be contracted by actual contact.

Dr. Biegler—I coincide with Dr. Hawley. A miasm is an imponderable force attacking that person that is ready for it; and this point is very important, as it will explain why one among ten individuals will be attacked by an epidemic and the rest not; and I consider that this idiosyncrasy is nothing but a derangement of the vital force. The more this is deranged, the more severe will be the result of the attack of the miasm, and this again explains why one may recover and the other die, and a third one will only partly recover.

If, for instance, malaria attacks a perfectly healthy person, a dose of China may be sufficient to restore him to health, but the reason why China hardly ever does so is, that there are so few perfectly healthy persons, that most of them carry a latent chronic miasm which starts into life when the body is attacked by any acute miasm. Therefore we cannot cure marsh miasm, or only very seldom, without antipsoric remedies. An example showing how disease dynamis can be carried about, happened in my own family. When my son was sick with diphtheria, a friend of mine, a physician from Brooklyn, called on me, saw the child, and seeing how sick he was, offered to stay. He was taken with sore throat the same night but returned next morning to my home, remained an hour, and then started for home. A week after he wrote that he buried his child, dying with the same kind of diphtheria that mine did.

Dr. Voak—I believe thoroughly in the statements made by Dr. Biegler, and believe that a perfectly healthy person can come in contact with these contagious diseases without contracting them.

Dr. Hawley—I think we will never get rid of contagion until all dead bodies are cremated. A flock of sheep, numbering thirty, was attacked by anthrax, of which twenty-eight died; these were buried in a corner of the pasture they had been kept in, and for the next seven years no sheep were put into this same pasture. After this lapse of time fifteen healthy sheep were brought into this field where the bodies of those dead from anthrax were buried, and in a short time all these fifteen sheep died from anthrax. He also mentioned that a table, old and rotten, which had been used formerly in a small-pox hospital to put bodies on, spread small-pox in all the families that used its wood for kindling. He further related a case of a lady, after confinement, who expected her mother-in-law to take care of her. Mother did not come, but sent explanatory letter, in which she stated that she had to help another daughter to take care of her children, sick with the measles. This letter was written in the sick-room. The lady who received the letter got the measles and died. A boy was transferred from an orphan asylum in Rochester, where scarlet fever was raging, to a Syracuse orphan asylum. Soon scarlatina broke out there, but the boy himself did not contract it. In one family a child was treated for scarlet fever; after its recovery the doctor ordered all the clothing and bedding burned. A year after, the mother gave birth to a child, and after some time this baby broke out with scarlet fever. There was no scarlet fever in the locality, but rigid research brought to light that the mattress which the child used that had the scarlet fever first had not been burned, according to the doctor's directions, but had been used for the new member of the family. Again this *corpus delicti* was condemned to destruction by fire. Two years later another baby was born, which after some time came down with scarlet fever, and again the old mattress turned up as the cause for this event; but this time it was really burned.

Dr. Schmitt—If the late Dr. R. R. Gregg's discovery of the ultimate life cell be true, and I suppose all of you received his pamphlet on this subject, everything about contagion and the spontaneous outbreak of epidemics, under certain favorable telluric and atmospheric changes could be easily explained. The doctor claimed that this ultimate life cell which he gained from the decomposition of fibrin, if I am right, was indestructible. He exposed it to furnace heat for a week, found it shriveled and dried up, but if exposed to moisture and a certain degree of warmth, it would again revive and be able to propagate itself. The same was the case after long exposure to a great degree of coldness.

Now this cell could also be the carrier of the dynamis of any miasm that had pervaded the body to which it once belonged, and after lying anywhere for years and years, would, if brought under favorable conditions which started it to new life, start also with its new life the dynamis of which it had been the bearer for so long a time. If we then would compare this cell with one of our sugar globules, which is the bearer of the dynamis of a medicinal force, how easy could we explain all those to all appearances insoluble phenomena that we encounter in the history of contagion, epidemics, and certain miasmatic diseases. Then, of course, cremation would not help us to destroy the miasms, but we should perhaps look closer for that medicinal dynamis which would enable us to fight the miasmatic dynamis.

Dr. Biegler would especially emphasize the point made by Dr. Schmitt, that the ultimate cell was only the bearer of the miasm and not the disease germ itself.

Dr. Hawley compared dynamization with an electrical current, which creates the same electricity in a wire running parallel with the wire charged with the electricity.

Dr. Biegler—We must understand the chronic miasms of Hahnemann before we can treat the acute miasms; if they are not taken into consideration it will lead the practitioner to use material doses. A professor of a college who had had diabetes for a month came under my treatment. I prescribed for the man according to Hahnemann, and not for diabetes, without much expectation, as the patient was pretty low and high in years. But he recovered anyway, after a single prescription. After being well for a few months, an ulcer broke out in one of his legs and he was taken to a hospital in Buffalo, where he was treated according to "scientific" principles, which resulted in his death a month later.

I want to ask the allopaths and their mimickers, why did he get well when he was treated according to the law *Similia similibus curantur*, and why did he die immediately under "scientific" trentment?

Dr. Brewster—Did you use diabetic diet?

Dr. Biegler—Yes; but I have never seen a case that was cured by it alone.

Dr. Hawley—That man was sick and one of his symptoms was that he had sugar in the urine, and I am sure no case can be cured by withholding sugar from the daily food, but if you cover the symptoms with the right homœopathic remedy, you will cure the patient. A chronic miasm underlies all such diseases, and unless a man gets rid of materialism he will never be able to cure his patients.

Dr. Brewster—A man with diabetes was not kept under diabetic diet, but received for his symptoms—among which "Oh! how my back aches!" was the most peculiar—Lac vaccinum defloratum, high. In a few weeks he felt better, and had eaten all the time, as he always did. The Doctor further asked whether we should look for the cause of diabetes in the liver, lungs, or brain.

Dr. Biegler—Whether we know this or not is of no importance for practical purposes, but in trying to answer this question we may lose ourselves in speculations and vain theories. As long as we have a law of cure, we ought to be satisfied in curing our patient.

Dr. Clapp—By experiment it has been proven that by injuring certain portions of the brain sugar in the urine has been produced.

Dr. Biegler—We know that an injury to any nerve-tissue will be followed by saccharine urine, but I have had diseases of the liver with sugar in the urine where there was no brain difficulty at all. It only shows we may take up such a subject and never end it.

Dr. Hawley prescribed Lac vaccinum deflorat.$^{cm}$ in a case of diabetes where there was an enormous quantity of urine voided every day and weakness and lassitude very predominant. Two doses of the medicine were given six weeks apart. Within a week the patient was able to work around in the house, and finally got well. The patient was old Dr. Schenck, of this Society. As to diet, I think a perfectly well man can digest almost anything, and a man must be sick if his food distress him. Therefore I allow my patients to eat anything they relish.

Dr. Brewster—A baby vomiting everything, and having withstood three allopathic physicians and the different kinds of drugged food they had ordered, as, for instance, milk with lime-water, wine, and laudanum, was put on diluted milk and the appropriate remedy, and got well.

Dr. Biegler—We should not become loose in our dietetics, especially in feeding children, and we should always remember that God put in a mother's breast milk, and not oat-meal gruel. People are generally too apt to begin too early to feed their children with starchy food and to withhold milk.

Dr. Carr related a case of diphtheria due to chronic poisoning from well-water. The patient, a young man, had been using the water from a certain well right along, although he had been breaking out into boils, and the working-men, who drank from it occasionally, had been invariably attacked with diarrhœa. This case of diphtheria had at first received local treatment, and died in spite of the most careful homœopathic prescribing. The mother, who took care of the patient, contracted the disease and soon got well under the appropriate remedy, so that the doctor ceased attendance. But, not satisfied with her rapid cure, she used Chloride of Potassa gargle, had a relapse and died.

Dr. Biegler—I have treated a great many cases of diphtheria, and patients have spit in my mouth, nose, and eyes, but never contracted the disease, which shows that if the idiosyncrasy is wanting there is no contagion. If I find a case that has b[illegible]n treated locally I refuse to treat him.

Dr. Stow mentioned an old-school physician who had even lost all faith in local treatment in diphtheria.

Dr. Hawley, as member of the Committee to report on the death of the late Dr. T. L. Brown, asked for further time. Granted. As Chairman of the Board of Censors, he reported the application of Dr. A. V. Hoard.

Dr. Biegler moved that the subject for discussion at the next meeting be miasms, acute and chronic, according to Hahnemann. Carried.

Dr. J. G. Schmitt reported, as Treasurer, a balance of four dollars and thirty-one cents. Report accepted.

Dr. Biegler—Before adjourning, I want to state the cause of the absence of Dr. E. P. Hussey, of Buffalo. One of his children is seriously sick from the sequelæ of scarlet fever.

Dr. Hawley moved that a resolution of sympathy for Dr. Hussey be passed, and the Secretary be requested to transmit such resolution to the Doctor. Carried.

The meeting adjourned, to reassemble in Dr. Hawley's office, at Syracuse, on the third Thursday in March, 1888.

JULIUS G. SCHMITT,
*Secretary.*

